# KMT2B and Neuronal Transdifferentiation: Bridging Basic Chromatin Mechanisms to Disease Actionability

**DOI:** 10.1177/2633105520928068

**Published:** 2020-06-14

**Authors:** Giulia Barbagiovanni, Michele Gabriele, Giuseppe Testa

**Affiliations:** 1 Department of Experimental Oncology, IEO, European Institute of Oncology IRCCS, Milan, Italy; 2 Department of Biological Engineering, Massachusetts Institute of Technology, Cambridge, MA, USA; 3 Department of Oncology and Haemato-Oncology, University of Milan, Milan, Italy; 4 Human Technopole, Milan, Italy

**Keywords:** Transdifferentiation, reprogramming, histone methylation, H3K4me3, KMT2A, KMT2B, MLL1, MLL2, GABA, murine, myelin, neurodegeneration, neurogenesis, neurogenetics, synaptogenesis

## Abstract

The role of *bona fide* epigenetic regulators in the process of neuronal transdifferentiation was until recently largely uncharacterized, despite their key role in the physiological processes of neural fate acquisition and maintenance. In this commentary, we describe the main findings of our recent paper “KMT2B is selectively required for neuronal transdifferentiation, and its loss exposes dystonia candidate genes,” where we investigated the role of this histone H3K4 methyltransferase during mouse embryonic fibroblasts (MEFs) to induced neuronal cells (iNs) direct conversion. Indeed, *Kmt2b*^–/–^ MEFs, transduced with three neuronal-specific transcription factors (TFs), *Brn2, Ascl1*, and *Myt1l*, show lower transdifferentiation efficiency, defective iN maturation, and augmented alternative cell fates acquisition, with respect to controls. Here, we went beyond the data, hypothesizing how KMT2B executes its fundamental role. In particular, we supposed that MYT1L, which has been proven to be fundamental for iN maturation and the switch-off of alternative cell fates, directly or indirectly needs KMT2B. Indeed, KMT2B could be important both to make MYT1L-target genes accessible, because MYT1L is not a pioneer TF and preferentially binds to open chromatin, and to activate MYT1L-downstream genes.

**Comment on:** Barbagiovanni G, Germain PL, Zech M, et al. KMT2B is selectively required for neuronal transdifferentiation, and its loss exposes dystonia candidate genes. *Cell Rep*. 2018;25(4):988-1001. doi:10.1016/j.celrep.2018.09.067. PubMed PMID: 30355503; PubMed Central PMCID: PMC6218204. https://pubmed.ncbi.nlm.nih.gov/30355503/

Neuronal transdifferentiation refers to the direct conversion of mouse embryonic fibroblasts (MEFs) into induced neuronal cells (iNs), thanks to the expression of three neuronal-specific transcription factors (TFs): *Ascl1, Brn2*, and *Myt1l* (BAM [Brn2 Ascl1 and Myt1l] factors).^1^ As fibroblasts and neurons derive from different germ layers, the epigenetic “barrier” between them is usually depicted as a particularly steep hill separating the valleys across Waddington’s landscape. Indeed, at the end of transdifferentiation, the iN transcriptome resembles its normal neuronal counterpart and does not cluster anymore with fibroblast transcriptome.^2,3^ Although 10 years have passed since the MEF-to-iN conversion was first accomplished, when we started this work very little was known on the chromatin regulatory and epigenetic changes necessary for transdifferentiation since most research papers mostly focused on transcriptomic reset underlying MEF-to-iN direct conversion.^2-5^ In our recent work, we focused on the chromatin regulation of this process, investigating the role of two histone H3 lysine 4 (H3K4) trimethylases, KMT2A and KMT2B, whose role during neuronal differentiation has been extensively studied, to probe their specific impact on neuronal transdifferentiation.

Our contribution can be summarized in two main key findings: (1) KMT2B, unlike KMT2A, is crucial for the MEF-to-iN conversion, both for the induction of transdifferentiation process per se and the maturation of the generated iNs; and (2) the molecular pathways underlying neuronal cell conversion, which we exposed through conditional ablation of *Kmt2b*, can illuminate the genetic basis of dystonia, a neurological disorder for which *KMT2B* mutations have been recently found as driving genetic leads.^6,7^

Our research started from the following founding questions: (1) How does the combination of few lineage-specific TFs orchestrate, at the molecular level, the rapid and complete epigenome resetting, which leads to the generation of functional iNs? (2) More specifically, what is the role of KMT2B during transdifferentiation and in which stages it is fundamental? To experimentally address these questions, we analyzed *Kmt2b*^–/–^ and controlled transdifferentiating cells before and along transdifferentiation, at both the chromatin and transcriptome level.

On one hand, by analyzing MEFs upon *Kmt2b* deletion, but prior to BAM transduction, we demonstrated that *Kmt2b*^–/–^ MEF epigenome is already reset before cell conversion. Indeed, many genes dysregulated along transdifferentiation in the absence of KMT2B, lose H3K4me3 just upon *Kmt2b* deletion. This effect is accompanied by an increased deposition of H3K27me3, which is maintained, to our knowledge, at least until the fifth day of transdifferentiation. As we were able to detect *Kmt2b*^–/–^ iNs at the end of transdifferentiation, it is worth hypothesizing that, even if *Kmt2b*^–/–^ MEF epigenome is different from the one of control MEFs, ASCL1 is anyway able to start transdifferentiation, thanks to its “nature” of pioneer TF.^3,4^ However, transdifferentiation efficiency is lower in the absence of KMT2B. Thus, also the activity of ASCL1 itself could be less effective in both starting and driving cell conversion. Wapinski and colleagues demonstrated that efficiency of neuronal transdifferentiation positively correlates with the percentage of trivalent chromatin states, which are specific sites in the transdifferentiating cell type, composed by H3K4me1, H3K9me1, and H3K27ac.^3^ As KMT2 proteins, beyond catalyzing H3K4 trimethylation, are also responsible for the deposition of H3K4me1, we analyzed whether we were able to identify differentially monomethylated H3K4 sites between *Kmt2b*^–/–^ and control MEFs. As we quite expected, we did not score a dysregulation in H3K4me1 deposition, because this mark is mainly deposited by KMT2C and KMT2D.^8,9^ However, to date, we cannot exclude that the deposition of H3K9me1 and H3K27ac is not been impaired in the absence of KMT2B. Indeed, the increase in H3K27me3 we observed could result in a reduction in H3K27 acetylation,^10^ thus disrupting the pattern of trivalent chromatin states, which makes MEFs more favorable to transdifferentiate. Therefore, we demonstrated that the deletion of *Kmt2b* leads both to an impaired H3K4me3 deposition and a defective maintenance of the mark throughout MEF proliferation, which implies that KMT2A is not able to compensate. Moreover, the disruption of H3K4me3 pattern influences the deposition of other epigenetic marks, such as H3K27me3. Thus, most likely, the epigenomic signature of *Kmt2b*^–/–^ MEFs is very different from that of control cells, and this difference is not confined to H3K4me3 and H3K27me3. This could explain both the less effective induction of transdifferentiation and the defective activation of pathways underlying neurogenic fate acquisition. Indeed, as mentioned above, we demonstrated that a portion of differentially expressed genes during transdifferentiation loses H3K4me3 already at MEF stage upon *Kmt2b* deletion.

However, the epigenome resetting in *Kmt2b*^–/–^ MEFs could not be the only cause of the defective transdifferentiation we observed in knockout with respect to control. Indeed, we showed that specific H3K4 *loci* lose trimethylation during transdifferentiation and that this loss is maintained and augmented during cell conversion, while only a small portion of them is compensated at the end of the process, most likely by KMT2A. Thus, KMT2B is fundamental both for the maintenance of the MEF epigenome, influencing MEFs propensity to transdifferentiate, and during transdifferentiation itself, allowing the activation of genes necessary to induce neuronal lineage and, in particular, the ones related to iN maturation.

Furthermore, we demonstrated that KMT2B is also fundamental to switch off alternative lineages. Indeed, integrating Wapinski and colleagues’ and our data, we showed that the genes upregulated in *Kmt2b*^–/–^ cells which fail transdifferentiation (ie, PSA-NCAM^–^ cells) are mainly related to myocyte fate acquisition.^3^ These genes are normally early upregulated during MEF-to-iN conversion driven both by *Ascl1*-alone and by BAM factors but are switched off later on, in the second case, when *Myt1l* and *Brn2* are co-transduced with *Ascl1*.^3^ Furthermore, we evidenced that most of these genes are bound by ASCL1 during the first phases of transdifferentiation.^3^ Mall and colleagues have demonstrated that, during MEF-to-iN conversion, MYT1L behaves mainly as a transcriptional repressor, downregulating both fundamental genes associated to the acquisition of alternative fates, especially the myocyte one and the genes that negatively regulate neuronal differentiation (eg, *Id1* and *Id3*).^2,3,11^ On the other side, a portion of MYT1L-target genes is upregulated during transdifferentiation. Interestingly, among the others, upregulated genes are related to “histone modification,” “chromosome organization,” and “generation of neurons” as gene ontology categories.^11^ Furthermore, also during physiologic neuronal specification, MYT1L upregulates proneural TF genes, thus modulating, for example, neurite outgrowth and synaptic transmission.^12^ Thus, MYT1L should lead to a dual action in transdifferentiating MEFs: (1) the acquisition of neural fate, through a fine-tuned activation and repression of specific neuronal pathways, and (2) the repression of alternative lineages. Therefore, because both iN maturation and suppression of alternative fates are impaired in the absence of KMT2B, we hypothesize that in these phases of transdifferentiation, MYT1L requires KMT2B to accomplish its role. On one side, KMT2B could be fundamental to activate the pool of MYT1L-induced targets, trimethylating H3K4 at promoters of these genes. But, on the other side, what could be the role of a transcriptional activator, KMT2B, in MYT1L-mediated gene repression? It has been demonstrated that, differently from ASCL1, during transdifferentiation, MYT1L preferentially binds to active promoters only, enriched for H3K4me3 and H3K27ac.^11^ Thus, it is worth hypothesizing that H3K4me3 loss and H3K27me3 enrichment in MEFs upon *Kmt2b* deletion could highly affect MYT1L binding, with consequences in MYT1L-mediated repression of its specific targets.

Moreover, we showed that *Zfp238*, a downstream target of ASCL1, fundamental to guarantee success of the transdifferentiation process,^3^ is upregulated upon *Ascl1* induction and maintained expressed until the end of MEF-to-iN conversion in iNs. In the absence of KMT2B, *Zfp238* is anyway upregulated at the beginning of transdifferentiation, confirming the pioneering nature of ASCL1. However, on the contrary of control cells, it remains expressed also in *Kmt2b*^–/–^ untransdifferentiated cells (ie, PSA-NCAM^–^ cells). ZFP238, during myogenic differentiation, can also be induced by MyoD and inhibits *Id2*/*Id3*, which in turn would have inhibited the TF *Myogenin*.^13^ Thus, ZFP238 allows MyoD-driven myogenic differentiation. Therefore, the fact that *Zfp238* remains expressed in untransdifferentiated cells, on one side, further confirms the stronger switch of *Kmt2b*^–/–^ cells toward the myocytic fate. On the other, it could explain why, maybe, we did not find *Id* genes dysregulated, despite we hypothesized that KMT2B is necessary for MYT1L to accomplish its role. In Figure 1, our hypothesized expanded mechanism of action is reported.

**Figure 1.**
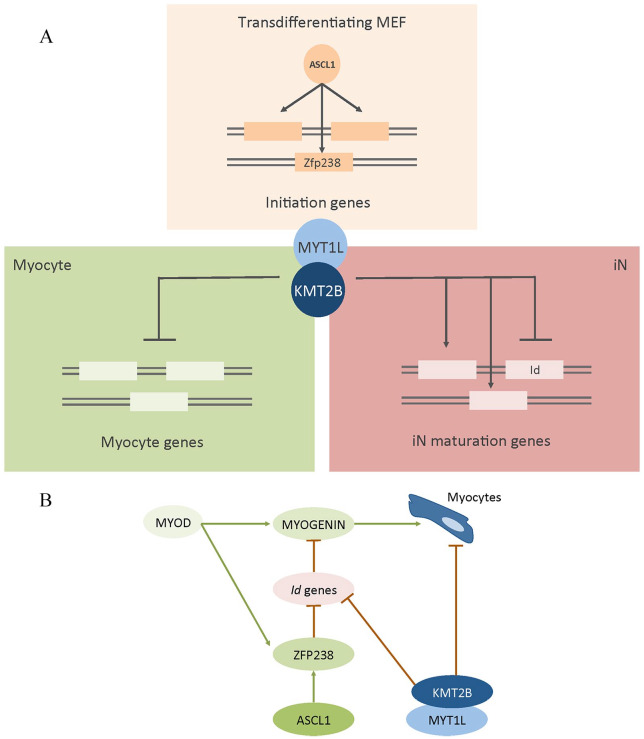
(A) Schematic representation of the hypothesized downstream pathways to ASCL1 and MYT1L during transdifferentiation. Also, the hypothesized KMT2B role is reported. (B) Schematic hypothesized myogenic pathway switched off by MYT1L during transdifferentiation. iN indicates induced neuronal cells; MEF, mouse embryonic fibroblasts.

Finally, through the analysis of transcriptomic changes upon *Kmt2b* deletion, we identified specific direct and indirect KMT2B targets, fundamental during neuronal specification. As mentioned above, it has been recently shown that specific mutations on *KMT2B* gene could lead to dystonia.^6,7^ However, because not all dystonia patients present a *KMT2B* mutation, we asked ourselves whether also a downstream disruption of the neuronal *KMT2B* pathway could lead to the disease. To assess this hypothesis, we investigated whether the exomes of dystonia patients had a mutation in some of the KMT2B targets identified thanks to the molecular dissection of KMT2B role during transdifferentiation. We found three promising candidates: *NOL4, SLC35F1*, and *SLC40A1*. In particular, *SLC40A1* has been found associated with hemochromatosis type 4, a disease highly related to iron deposition.^14,15^ Indeed, when we went back to the patient, we found that he or she had elevated serum-ferritin and possibly iron deposits on brain magnetic resonance imaging (MRI; because he had bilateral signal alterations of basal ganglia). To date, signal alterations of basal ganglia described in most KMT2B-mutated patients had never been associated with iron deposits. Importantly, however, an iron-lowering therapy improved the condition of this patient, in what we found a particularly rewarding twist of how a squarely foundational line of research can disclose mechanisms and therapeutic inroads that can have significant and at times also very accelerated impacts on patients’ conditions.

In sum, we reflect on our article as an example of mechanistic neurobiology that highlights the following main features: (1) first, the value of dissecting the non-physiological, experimental acquisition of cell fate in illuminating very selective roles that highly homologous chromatin regulators play in specific neuronal properties and that had not been uncovered in the study of physiological neurogenesis; (2) second, the translational value of such mechanistic insights when productively applied to phenotypically well-characterized disease cohorts to illuminate the pathogenic significance of genetic variants including their actionability in current paradigms of care.
